# A solitary urothelial tumor arising from one of bilateral ureteroceles

**DOI:** 10.1590/S1677-5538.IBJU.2017.0087

**Published:** 2017

**Authors:** Yu Xi Terence Law, Irfan Khan Sagir, Lincoln Tan Guan Lim

**Affiliations:** 1 Department of Urology, National University Hospital, National University Health System, Singapore; 2 Department of Pathology, National University Hospital, National University Health System, Singapore

## INTRODUCTION

A ureterocele is a congenital defect of the ureter. Based on available autopsy reports, the highest incidence of ureteroceles has been reported as 1 in 500, occurring four to six times more commonly in females than in males. It can occur in up to 95% of females with a duplex collecting system (1, 2).

Ureteroceles in adults usually arise within a single renal system with no or mild obstruction. They may present as recurrent urinary tract infections, flank pain or remain asymptomatic, only to be picked up incidentally via imaging. Stasis and infection may predispose to calculus formation in the ureterocele and upper urinary tract (3).

The association of a ureterocele with a tumor is very uncommon. In this paper, we report a unique case of a tumor arising within one of bilateral ureteroceles.

### Case Presentation

**Figure 1:**
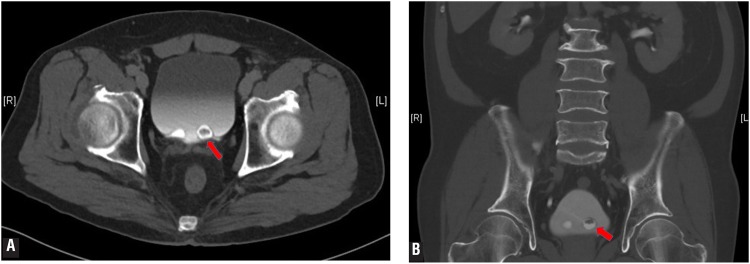
A and B) CT Urography revealed bilateral simple ureteroceles with the left containing a slightly enhancing soft tissue nodule measuring 0.4 × 0.7 × 0.9cm (red arrow).

**Figure 2:**
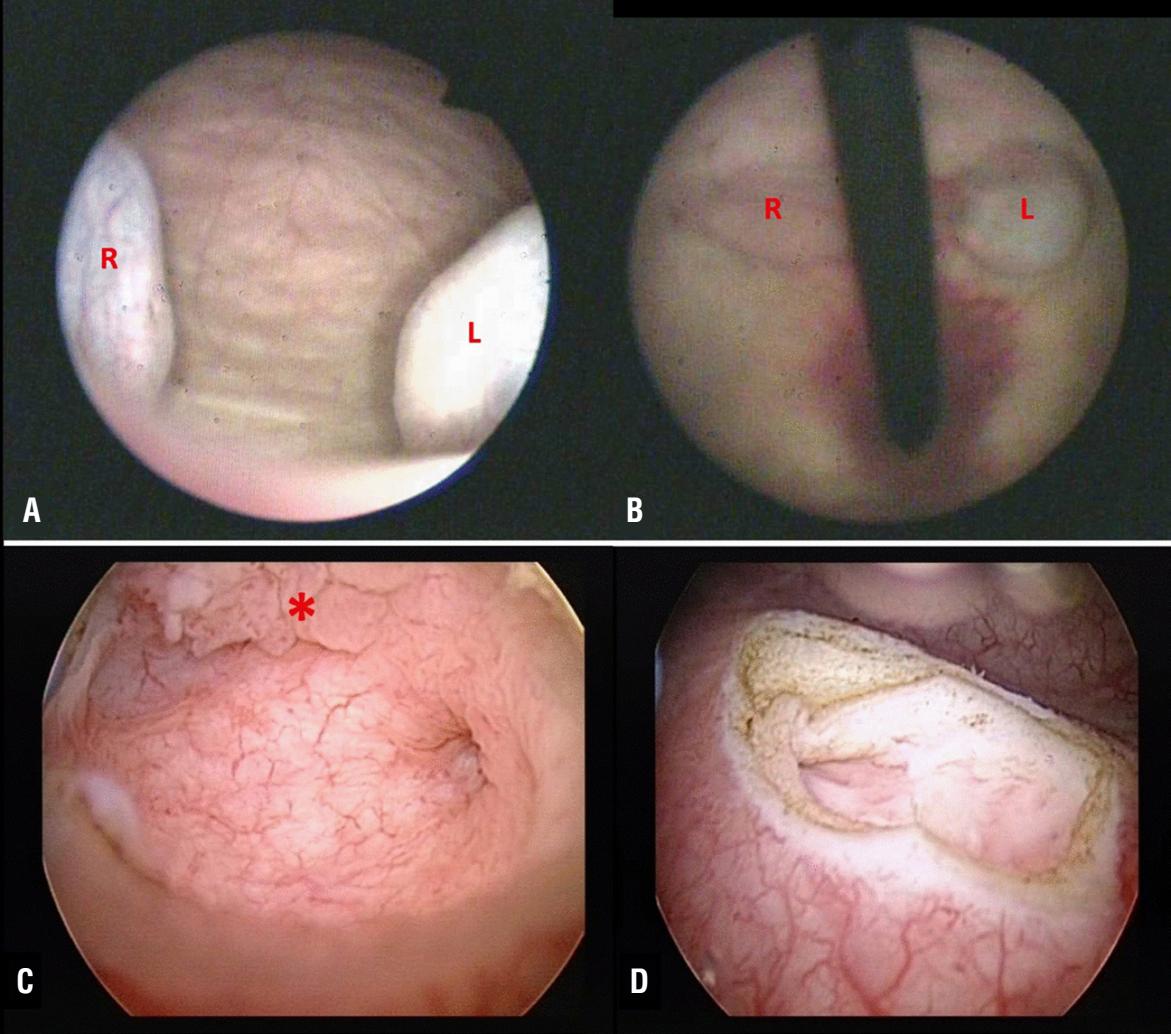
A and B) Flexible cystoscopy was done and bilateral ureteroceles were seen but no suspicious lesion was seen as the lesion was enclosed within the left ureterocele (R- Right, L- Left). C) There is a papillary lesion on the inner surface sparing the proximal end of the ureterocele and the ureteric orifice (red asterisk). D) The right ureteric orifice was normal.

A 67-year-old Chinese male presented with painless gross hematuria after sexual intercourse. He was a non-smoker, and had no previous contact with anilines or other chemicals. Urine cytology and serum prostate specific antigen were normal. Computed tomography (CT) urography revealed bilateral simple ureteroceles with the left containing a slightly enhancing soft tissue nodule (Figures 1A and B). Flexible cystoscopy revealed bilateral ureteroceles with no lesion seen. (Figures 2A and B).

Transurethral unroofing of the left ureterocele containing the tumor together with right ureterocele was performed. There was a papillary lesion on the inner surface sparing the proximal end of the ureterocele and the ureteric orifice (Figure-2C). This corresponded to the filling defect that was visualised on CT urography. The right ureteric orifice was normal (Figure-2D). The patient was elected for resection of both ureteroceles. This was to prevent the development of metachronous tumors and complications such as calculi formation and infection, as well as to simplify cystoscopy surveillance.

**Figure 3:**
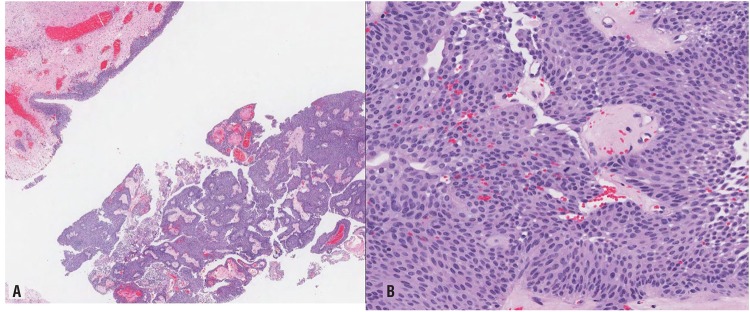
A) Photomicrograph showing tumour cells which display round to oval nuclei in a papillary architecture with fibrovascular cores, no stromal invasion seen (H&E x 20). B) The tumour cells exhibit mild nuclear atypia and occasional mitosis, no high grade nuclear features present (H&E x 200).

The pathology laboratory received multiple fragments of tissue, aggregating 1.5x1.5x0.3cm and weighing 0.5g. Histological examination of resected specimen revealed tumor cells displaying round to oval nuclei arranged to form a papillary architecture with fibrovascular cores, without stromal invasion. The tumor cells exhibited mild nuclear atypia and occasional mitoses, and there were no high grade nuclear features seen. These findings were consistent with a low-grade noninvasive papillary urothelial carcinoma (Figures 3A and B). Post-operatively, the patient was given 40mg intra-vesical mitomycin. To date, this patient is recurrent free 3-months post resection. His cystoscopy surveillance schedule will be as for low risk bladder transitional cell carcinoma following the American Urological Association guidelines (4).

## DISCUSSION

A combination of imaging techniques for ureteroceles has been employed in the past till present. Modalities include ultrasonography, intravenous pyelogram (IVP) and CT urography. Classical appearances of ureteroceles based on different imaging modalities are described below.

The sonographic finding of a well-defined cystic intra-vesical mass within the posterior bladder wall is suggestive of a ureterocele and a classic description is that of a cyst within a cyst. Tumors arising from the ureterocele may bear features of irregular echogenicity without acoustic shadowing (5). However, these may be missed if the patient's bladder is empty or fully distended, or if the ureteroceles were small.

The classic finding on IVP is a round radiopacity in the bladder surrounded by a radiolucent rim. The characteristic appearance of a ureterocele on CT urography is an intra-vesical defect that is radiolucent and globular in nature manifesting as the “cobra-head sign” (5). The CT urogram is useful in visualizing enhancing masses such as tumors. It also excludes extra-vesical disease. While CT urography has gradually replaced the use of IVP in more recent times, each of the three above mentioned techniques demonstrate some utility in the initial imaging of a ureterocele.

Magnetic resonance imaging (MRI) is usually not used, but it should be as effective as IVP and CT urogram for visualization of ureteroceles, especially when MR urography is performed with or without contrast (6). There is limited data on how MRI may be useful for imaging tumors which arise from these ureteroceles.

There are fifteen reports of tumors arising from true ureteroceles [5, 7–12], however this is the first report of transitional cell carcinomas (TCC) in a patient with bilateral ureteroceles. In a true ureterocele TCC represent the majority of tumors that may arise [5, 7, 8, 10–12], although one case of squamous cell carcinomas has been reported (9). This is because the urothelial tissue preserves its capacity to undergone malignant transformation (13). Regarding imaging findings, these 15 reports detail similar findings as described above. Although cystoscopy successfully demonstrated the presence of tumors encroaching on the outer surface of the ureterocele in 3 out of 15 of the cases (5, 10), cystoscopy in our case did not reveal any suspicious features arising from the left ureterocele as the tumor was enclosed within the inner surface of the ureterocele. Hence there was a need for transurethral unroofing of the ureterocele for visual confirmation and resection for clearance and histological diagnosis of the tumor.

This case highlights the need for upper tract imaging as an investigation for gross hematuria regardless of whether urine cytology reflects no evidence of malignancy. Common causes of gross hematuria include urinary tract infections, stones and malignancies. Whilst highly unusual, this patient was found to have bilateral ureteroceles, of which one harbored a tumor. There are no current guidelines on the management of urothelial tumors arising from ureteroceles. However, there is general consensus that when the tumor does not encroach the ureteric orifice or distal ureter, adjuvant therapy and intensity of surveillance can be guided by the histological grade and stage of the tumor (4).
